# Artificial intelligence in diagnosing dens evaginatus on periapical radiography with limited data availability

**DOI:** 10.1038/s41598-023-40472-3

**Published:** 2023-08-14

**Authors:** Eunhye Choi, KangMi Pang, Eunjae Jeong, Sangho Lee, Youngdoo Son, Min-Seock Seo

**Affiliations:** 1 School of Dentistry, Dental Research Institute, Seoul National University, Seoul, Republic of Korea; 2 Department of Oral and Maxillofacial Surgery, Seoul National University Dental Hospital, Seoul, Republic of Korea; 3 Department of Industrial and Systems Engineering, Dongguk University - Seoul, 30 Pildong-ro 1-gil, Jung-gu, Seoul, 04620 Republic of Korea; 4 Data Science Laboratory (DSLAB), Dongguk University - Seoul, Seoul, Republic of Korea; 5 Department of Conservative Dentistry, Wonkwang University Daejeon Dental Hospital, 77 Dunsan-ro, Seo-gu, Daejeon, Republic of Korea

**Keywords:** Dental pulp, Computer science

## Abstract

This study aimed to develop an artificial intelligence (AI) model using deep learning techniques to diagnose dens evaginatus (DE) on periapical radiography (PA) and compare its performance with endodontist evaluations. In total, 402 PA images (138 DE and 264 normal cases) were used. A pre-trained ResNet model, which had the highest AUC of 0.878, was selected due to the small number of data. The PA images were handled in both the full (F model) and cropped (C model) models. There were no significant statistical differences between the C and F model in AI, while there were in endodontists (*p* = 0.753 and 0.04 in AUC, respectively). The AI model exhibited superior AUC in both the F and C models compared to endodontists. Cohen’s kappa demonstrated a substantial level of agreement for the AI model (0.774 in the F model and 0.684 in C) and fair agreement for specialists. The AI’s judgment was also based on the coronal pulp area on full PA, as shown by the class activation map. Therefore, these findings suggest that the AI model can improve diagnostic accuracy and support clinicians in diagnosing DE on PA, improving the long-term prognosis of the tooth.

## Introduction

Dens evaginatus (DE) is a rare developmental anomaly, characterized by abnormal tooth development, resulting in the projection of an extra cusp or tubercle on the occlusal surface of posterior teeth and the lingual surface of anterior teeth^1^. The mandibular premolars are the most frequently observed DE^2^. This cusp-like protrusion is covered by an enamel layer that contains a dentin core and a thin extension of pulp, which makes it susceptible to pulpal complications from wear or fracture^3^. Patients with moderate to severe DE may experience complications such as tooth fracture, pulp exposure, pulp necrosis, and periapical; pathosis^4^. To prevent this complications, several treatment options were reported including intermittent grinding or tubercle protection using filling materials^3^ and early diagnosis is necessary.

The prevalence of DE has been estimated to range from 0.5 to 4.3%, and it may be influenced by a combination of genetic and environmental factors, with individuals of Mongoloid origin exhibiting a higher incidence of this condition. Specifically, among Chinese, Japanese, Malays, Filipinos, certain Eskimo populations, American Indians, and Aleuts, the reported prevalence is up to 4.3%^2,5^. The rarity of DE makes it challenging to obtain substantial image data on this anomaly, especially in East Asian populations, where its prevalence is less than 4%.

Periapical radiography (PA) is a widely used imaging modality that provides detailed images of a single tooth and its surrounding structures with improved resolution compared to that of dental panoramic radiography. Although the anatomical characteristics of DE can be visually determined in the oral cavity when the tubercle is well maintained, it may become increasingly difficult to distinguish as the tubercle wears down over time. However, the projection of the pulp along the protruding outline can still be observed using PA, and it could be easily damaged due to its thin suprastructures.

It is crucial to have accurate methods for diagnosing DE, and artificial intelligence (AI) models have demonstrated excellent performance in mimicking the precision and accuracy of trained dental specialists^6–9^. The use of AI in endodontics, including for periapical lesion^10^, vertical root fracture^11^, tracing of the apical foremen^12^, and detection of impacted mesiodens^13^, has demonstrated accurate diagnosis using PA^14^. However, there have been no studies focused on DE due to its rarity. With the increasing availability of large medical image databases, AI has the potential to learn from and analyze vast amounts of data, thereby achieving improved accuracy over time. The low prevalence of some medical conditions can make it challenging to collect data for deep learning (DL) model development, thus making it necessary to focus on clinically significant cases.

This study aimed to develop an AI model for accurately diagnosing DE using PA, particularly focusing on the cases with potential complications. We compared the diagnostic performance of the AI model using full and cropped images and assessed its accuracy compared to endodontists’ determinations. Two null hypotheses were tested in this study: (1) there is no difference in diagnostic accuracy between the AI model trained with the full-sized images and that with the cropped images, and (2) there is no difference in diagnostic accuracy between the AI model and endodontists’ diagnoses.

## Methods

### Materials

This study was approved by the Institutional Review Board (IRB) of Wonkwang University Daejeon Dental Hospital (W2204/1-1) and conducted in compliance with the approved ethical guidelines and regulations. The IRB approved a request to waive the documentation of informed consent for this retrospective chart review study.

The PA images for analysis were retrospectively selected from a database of dental images belonging to patients who visited the Department of Conservative Dentistry at Wonkwang University Daejeon Dental Hospital between March 2015 and February 2022. PA images were taken with intraoral X-ray unit (ProX; Planmeca, Helsinki, Finland) using a film sensor (RVG6200; Carestream, Rochester, NewYork, USA). The inclusion criteria for the classification as DE were as follows: 1) absence of caries, restorations, and periodontal problems; 2) presence of periapical lesion or symptoms of pulpitis; and 3) abnormal cusps observed by the clinician on the occlusal surface. In total, 138 DE images were obtained, including images of 19 (13.8%) mandibular first premolars and 119 (86.2%) mandibular second premolars, with a right-sided ratio of 50%. The proportion of females patients was 55.6% and the mean age was 14.9 years. The criteria for classification as normal were the absence of the DE criteria. In total, 264 normal images were obtained, including images of 54 (20.5%) mandibular first premolars and 210 (79.5%) mandibular second premolars, with a right-sided ratio of 58.4%. The proportion of female patients was 47.4% and the average age was 19.3 years.

### Methods

The study aimed to determine the most appropriate DL model for diagnosing DE in PA with a limited dataset. Five popular DL models in image classification, including a simple convolutional neural network (CNN) model, visual geometry group (VGG), densely connected convolution networks (DenseNet), residual neural network (ResNet), and inception-ResNet V2 (InceptionResNetV2), were selected, and their performances were evaluated based on the Area Under the Curve (AUC) metric.

The dataset was divided into a training set and a test set in an 8:2 ratio, respectively, and data augmentation was performed to enhance the model’s robustness. The performance of each model was evaluated with ten iterations of 50 epochs.

In the initial experiment, the full PA images were used in their original format (224 × 224 pixels), and the dataset was randomly split into 87.6% (352/402) for training and validation and 12.4% (50/402) for testing. To enhance the robustness of the model, data augmentation was performed using image rotation within ± 30 degrees, horizontal flipping, and brightness adjustment from 20 to 80% for each mini-batch in the training phase. The model was trained for 50 epochs using augmented data with a learning rate of 1.0 × 10^−4^ and an Adam optimizer.

In the second experiment, we used ResNet, which achieved the best performance in terms of the AUC metric in the first experiment. To further enhance the model performance, the image of the tooth of interest (first or second premolar) in the PA was cropped for analysis and compared with the performance of the model using the uncropped image. To ensure the model to be validated using all parts of data and avoid overfitting to a specific testing dataset, we used the cross-validation technique. Specifically, we employed the stratified $\mathrm{K}$-fold cross-validation to consider the limited amount of data and the class imbalance. Here, we set $\mathrm{K}$ to five. In addition, we explored optimal hyperparameters for image size, dropout rate, and learning rate, which were used to train the model, ranging in {(128, 128), (224, 224), (512, 512)}, {0, 0.1, 0.2, 0.3}, and {0.001, 0.0005, 0.0001, 0.00005}, respectively, by evaluating the model performance on the validation dataset. Consequently, we set the image size to (224, 224), the learning rate to 0.0001, and the dropout rate to 0.2. Moreover, the data augmentation procedure remained unchanged, in the second attempt.

**Figure 1:**
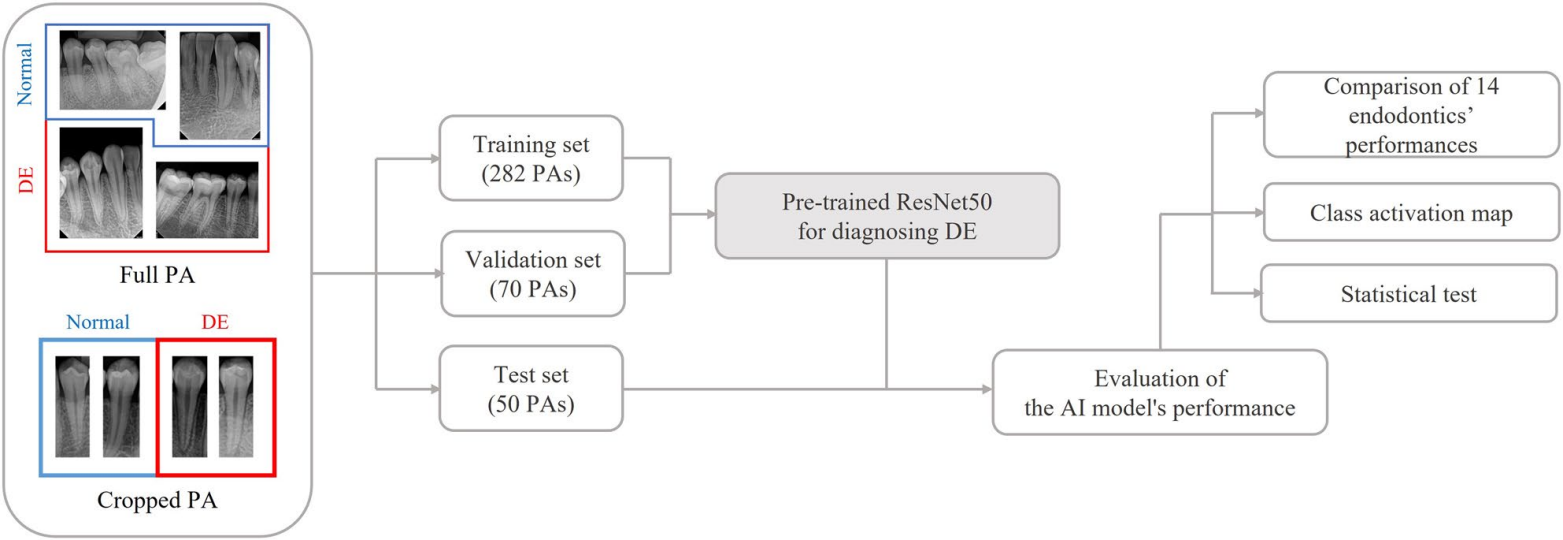
Flow of AI model development. 1. Collect and preprocess periapical radiography (PA) images. 2. Label images as either normal or showing dens evaginatus (DE). 3. Split images into training and testing datasets. 4. Use a pre-trained ResNet model as the basis for an artificial intelligence (AI) model. 5. Train the AI model using the training dataset. 6. Evaluate the AI model’s performance using the testing dataset, and compare it to endodontists’ performance. 7. Use a class activation map to determine the AI model’s judgment based on the coronal pulp area. 8. Analyze the statistical significance of the results using t-tests. AI, artificial intelligence; DE, dens evaginatus; PA, periapical radiography.

As a result, AI models were developed based on how the PA images were handled (Fig. 1):

1. The F model used full PA images (1876 × 1402 pixels) in their original format and resized (224 × 224 pixels)
2. The C model used cropped PA images (425 × 1005 pixels) that focused on the first or second premolar tooth and resized (224 × 224 pixels).

The class activation map was used to interpret the F model and visualize the regions of the image considered by the AI while making predictions. To assess the clinical feasibility of the AI, the results of the PA readings by the AI were compared to the determinations made by fourteen specialists (M:F = 4:10, mean clinical experience = 10.8 ± 5.1 years, range 4–18 years) in conservative dentistry who were not involved in this study. The same test set was extracted and evaluated by endodontists based on the highest AUC for both the F and C models.

### Model and statistical analysis

Accuracy, precision, recall, F1 score, and AUC were calculated to assess the performance of each model and endodontists. Accuracy represents the proportion of correct predictions, precision is the ratio of true positives to the sum of true and false positives, recall is the ratio of true positives to the sum of true positives and false negatives, the F1 score is the harmonic mean of precision and recall (i.e., (2 × precision × recall)/(precision + recall)), and AUC is the area under the Receiver Operating Characteristic curve. Cohen’s kappa was calculated to estimate the strength of agreement. Independent t-tests were conducted to compare the average diagnosis performances of F and C models of AI as well as those of the AI models and the experts, and paired t-test were used to compare the F and C models of the experts. *p* < 0.05 was considered statistically significant.

The analysis was performed using the Python programming language (version 3.8.5), Tensorflow (version 2.5.0), and a graphics card (GeForce RTX 3090; NVIDIA Corporation, Santa Clara, CA, USA).

### Ethics approval

The study was conducted in accordance with the guideline of the Declaration of Helsinki. Ethical approval was obtained by the Institutional Review Board of Wonkwang University Daejeon Dental Hospital (W2204/1-1). All methods were performed in accordance with the relevant guidelines and regulations. Participant consent was not necessary for this retrospective register study.

### Consent to participate

The IRB approved a request to waive the documentation of informed consent for this retrospective chart review study.

## Results

**Figure 2:**
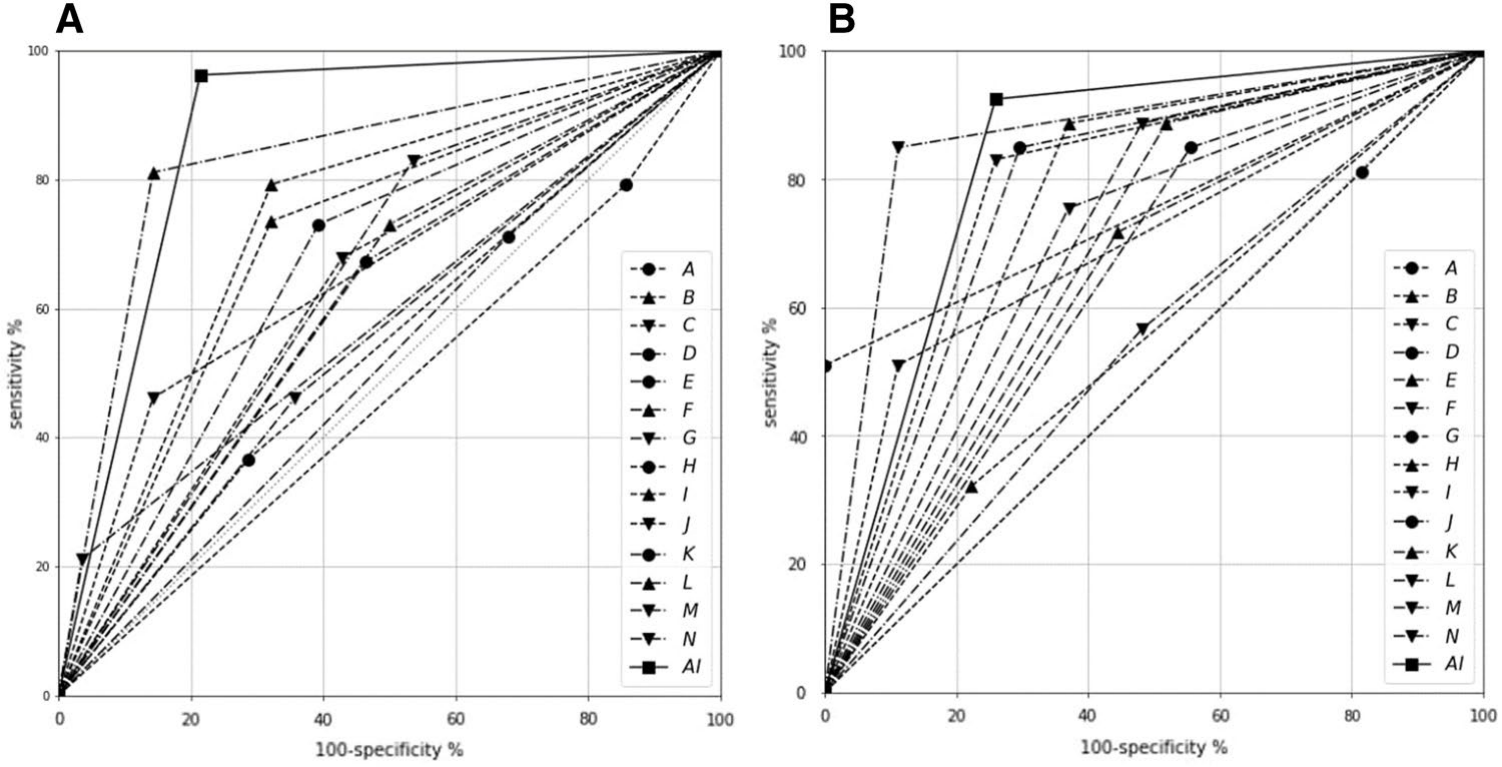
Comparison of sensitivities and specificities of fourteen endodontic specialists and the AI model for diagnosing DE on PA. (**A**) The F model used full PA images (1876 × 1402 pixels) in their original format and resized (224 × 224 pixels). (**B**) The C model used cropped PA images (425 × 1005 pixels) that focused on the first or second premolar tooth and resized (224 × 224 pixels). AI, artificial intelligence; DE, dens evaginatus; PA, periapical radiography, A-N, endodontic specialists.

Among the five DL models, ResNet showed the best performance, with an AUC of 0.878 (Table 1). The AI model was tested with five-fold cross-validation, and the F model had an average accuracy of 0.828, precision of 0.869, recall of 0.871, F1 score of 0.869, and AUC of 0.895. The C model had an average accuracy of 0.832, precision of 0.856, recall of 0.898, F1 score of 0.876, and AUC of 0.901 (Table 2). The AUC values of the F and C models were not found to be statistically different (*p* = 0.753; Table 3). For comparison, AI outperformed specialists in accuracy, precision, recall, F1 score, and AUC, regardless of the image processing method used (Table 4), as shown in Fig. 2. The average AUC value of the specialists was slightly higher in the C model than it was in the F model (0.633 vs 0.679), and there was a statistically significant difference in AUC (*p* = 0.040; Table 2). Cohen’s kappa demonstrated a substantial level of agreement for the AI model (0.774 and 0.684 in the F model and C model, respectively) along with fair agreement for specialists (0.238 and 0.359 in the F and C model, and 0.359), as can be seen in Table 4.

**Table 1:** Model performance among five DL models.

	Accuracy	Precision	Recall	F1 score	AUC
Basic CNN	0.675	0.749	0.782	0.757	0.736
ResNet	0.805	0.824	0.897	0.858	0.878
Res2	0.645	0.787	0.685	0.689	0.665
DenseNet	0.668	0.784	0.733	0.723	0.754
VGG	0.812	0.857	0.862	0.857	0.870

*DL* deep learning, *AUC* area under the ROC curve.

**Table 2:** Five-fold cross validation in the F and C models.

Work	Accuracy	Precision	Recall	F1 score	AUC
The F model, five-fold cross-validation with full PA images					
0	0.901	0.895	0.962	0.927	0.923
1	0.840	0.917	0.830	0.871	0.895
2	0.863	0.887	0.904	0.895	0.930
3	0.800	0.849	0.849	0.849	0.901
4	0.738	0.796	0.811	0.804	0.827
Average	0.828	0.869	0.871	0.869	0.895
The C model, five-fold cross-validation with cropped PA images					
0	0.813	0.852	0.868	0.860	0.922
1	0.825	0.831	0.925	0.875	0.883
2	0.863	0.875	0.925	0.899	0.900
3	0.850	0.873	0.906	0.889	0.906
4	0.810	0.849	0.865	0.857	0.895
Average	0.832	0.856	0.898	0.876	0.901

*PA* periapical radiography, *AUC* area under the ROC curve.

**Table 3:** Results of T-test for model performance comparison.

Model		Accuracy	Precision	Recall	F1 score	AUC
F and C models in AI	T-statistic	0.130	− 0.568	0.862	0.297	0.326
	*p*-value	0.900	0.586	0.414	0.774	0.753
F and C models in specialists	T-statistic	4.797	2.866	3.849	3.328	2.286
	*p*-value	0.000	0.013	0.002	0.005	0.040
AI and specialists in the F model	T-statistic	13.406	21.331	7.150	10.550	− 6.546
	*p*-value	0.000	0.000	0.000	0.000	0.000
AI and specialists in the C model	T-statistic	14.032	18.851	8.755	13.566	− 4.236
	*p*-value	0.000	0.000	0.000	0.000	0.001

*AI* artificial intelligence, *AUC* area under the ROC curve.

**Table 4:** Comparison of diagnostic performance across specialists and AI.

Reader	Accuracy	Precision	Recall	F1 score	AUC	Cohen’s Kappa	Kappa index
Experiment 1, five-fold cross-validation with whole PA images							
A	0.560	0.727	0.500	0.706	0.583	-0.073	Poor
B	0.716	0.813	0.736	0.772	0.707	0.398	Fair
C	0.600	0.857	0.462	0.600	0.659	0.266	Fair
D	0.625	0.729	0.673	0.700	0.604	0.202	Fair
E	0.575	0.661	0.712	0.685	0.516	0.034	Slight
F	0.650	0.731	0.731	0.731	0.615	0.231	Fair
G	0.475	0.917	0.212	0.344	0.588	0.132	Slight
H	0.488	0.704	0.365	0.481	0.540	0.066	Slight
I	0.753	0.824	0.792	0.808	0.736	0.463	Moderate
J	0.642	0.750	0.679	0.713	0.610	0.241	Fair
K	0.688	0.776	0.731	0.752	0.669	0.330	Fair
L	0.827	0.915	0.811	0.860	0.834	0.636	Substantial
M	0.704	0.746	0.830	0.786	0.647	0.310	Fair
N	0.525	0.706	0.462	0.558	0.552	0.091	Slight
Average	0.630	0.775	0.621	0.678	0.633	0.238	Fair
AI	0.901	0.895	0.962	0.927	0.874	0.774	Substantial
Experiment 2, five-fold cross-validation with cropped PA images							
A	0.600	0.662	0.811	0.729	0.498	-0.004	Poor
B	0.800	0.825	0.887	0.855	0.758	0.536	Moderate
C	0.638	0.900	0.509	0.651	0.699	0.329	Fair
D	0.713	0.750	0.849	0.796	0.647	0.313	Fair
E	0.663	0.760	0.717	0.738	0.636	0.265	Fair
F	0.713	0.800	0.755	0.777	0.692	0.374	Fair
G	0.675	1.000	0.509	0.675	0.755	0.412	Moderate
H	0.475	0.739	0.321	0.447	0.549	0.077	Slight
I	0.800	0.863	0.830	0.846	0.785	0.561	Moderate
J	0.800	0.849	0.849	0.849	0.776	0.553	Moderate
K	0.750	0.770	0.887	0.825	0.684	0.397	Fair
L	0.863	0.938	0.849	0.891	0.869	0.706	Substantial
M	0.763	0.783	0.887	0.832	0.703	0.433	Moderate
N	0.550	0.698	0.566	0.625	0.458	0.076	Slight
Average	0.700	0.810	0.730	0.753	0.679	0.359	Fair
AI	0.863	0.875	0.925	0.899	0.833	0.684	Substantial

*PA* periapical radiography, *AI* artificial intelligence, *AUC* area under the ROC curve, *A-N* endodontic specialists.

**Figure 3:**
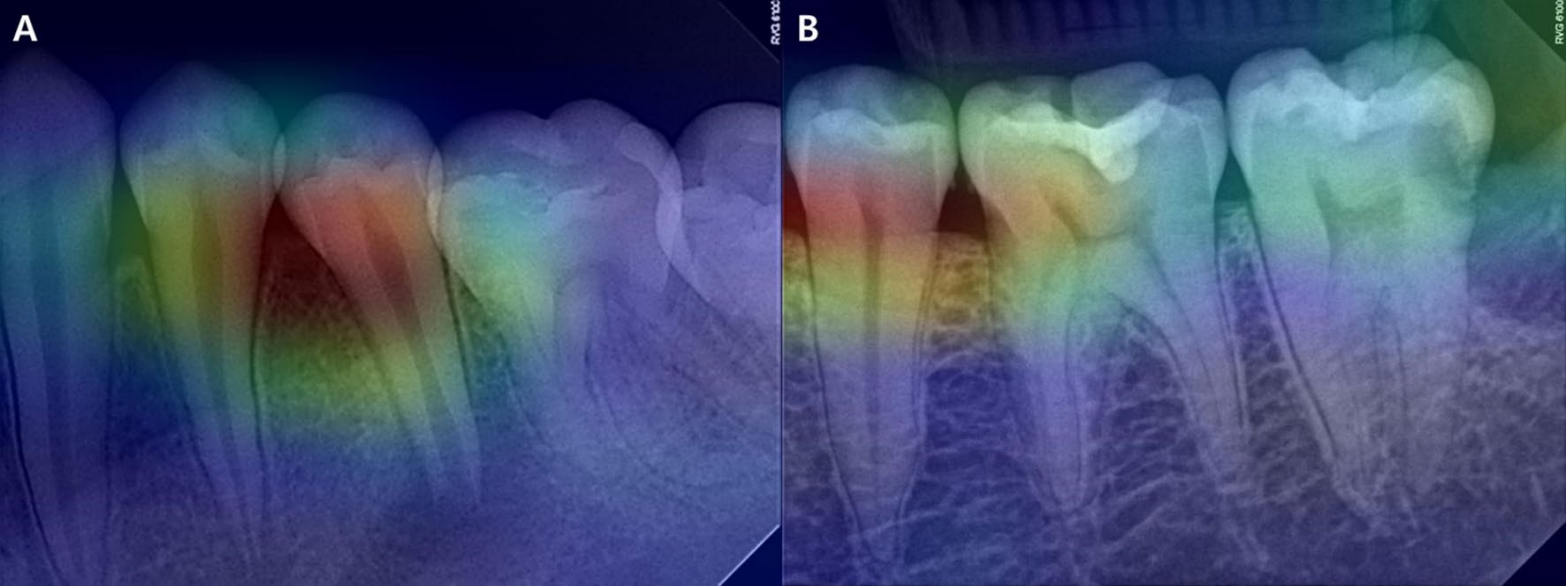
The class activation map in the F model showed that the AI model was focused on the coronal pulp area of the tooth during the decision-making process. (**A**) DE is visible on the mandibular second premolar as indicated by the white arrow, which points to the bulged border of the pulp roof in the PA. (**B**) Normal mandibular second premolar. AI, artificial intelligence; DE, dens evaginatus; PA, periapical radiography.

The class activation map in the F model showed that the AI model was focused on the coronal pulp area of the tooth during its decision-making process (Fig. 3).

## Discussion

This study developed an AI model for diagnosing DE on PA, which was shown to achieve higher accuracy than human specialists regardless of image cropping. DE is an observable morphological abnormality. However, distinguishing it becomes increasingly difficult as the tubercle wears down over time. Despite this, the projection of the pulp along the protruding outline can still remain, making it susceptible to damage due to its thin suprastructures. Additionally, clinicians may miss it if they are not paying attention. Applying the results of this study, AI can recognize it if PA was taken, enabling clinicians to choose preventive treatment options to avoid tooth damage.

PA is an essential diagnostic tool that can help clinicians identifying DE, assessing its size and shape, and evaluating the extent of periapical pathology. In some cases, additional diagnostic procedures such as cone-beam computed tomography (CBCT) may be needed to evaluate the complex root canal morphology and extent of periapical pathology, however, whether taking CBCT or not would be determined by PA. Through early diagnosis and management of DE, clinicians can increase the success rate of root canal treatment and improve the long-term prognosis of the tooth^4^. Among the treatment methods, regenerative endodontic procedures (REP) have demonstrated promising results in the treatment of teeth with DE and pulp necrosis^15^. REP is a biologically-based procedure that is designed to physiologically replace damaged tooth structures, including dentin and root structures, as well as cells of the pulp-dentin complex. However, REP are suitable for cases where the dental pulp is mildly to moderately affected or necrotic, rather than severely damaged. Therefore, the early recognition of DE allows for treatment choices that generally lead to good outcomes and can aid in the preservation of developing teeth in young patients^4^.

This study showed that AI could discern DE from normal cases more accurately than endodontist. Several studies reported similar results. Compared to expert clinicians, deep learning has shown highly accurate results with a sensitivity of 0.925 and specificity of 0.852 in identifying periapical radiolucent lesions in dental radiographs^16^. We found a similar sensitivity (0.943) and inferior specificity (0.763) when comparing AI to specialists. We believe that the latter is a more challenging topic for AI research, because finding a periapical lesion involve recognizing radiolucent changes in normal periodontal tissues that look radiopaque, while finding a DE involves noticing changes in appearance such as protrusion of the pulp area with radiolucency. In our previous study, AI could determine the bucco-lingual position of the inferior alveolar nerve relative to the mandibular third molar more accurately than specialists, where there was no effective method to discern. Sensitivity and specificity of AI were 0.867 and 0.75, while those of humans were much lower^7^. In this study, we were able to develop an AI model with higher sensitivity and specificity as a screening tool.

The choice of whether to use cropped or full images in an artificial intelligence (AI) model for PA did not affect the accuracy of the model. Cropped images can provide a more focused view of the region of interest, thus making it easier for the model to capture and analyze specific features. This can be advantageous in cases where the region of interest, such as a specific tooth or area of pathology, is small and difficult to discern in the full image. However, using cropped images can also result in a loss of context and important features that may be present in the surrounding structures. By contrast, full images provide a more comprehensive view of the entire region of interest, including the surrounding structures. This can provide additional contextual information that can be helpful in making a diagnosis. However, full images may also include extraneous information, such as overlapping teeth or artifacts, which can make it more difficult for the model to capture and analyze specific features. Unlike our expectation, our results showed no statistical differences between cropped images and full images. Similarly, in Matsuyama et al.’s study of pneumonia classification models, although the segmented images obtained higher accuracy than the original images, the segmented images can lead to erroneous resulting because AI may focus on the background region without structures and this may cause a disturbance in the prediction, in turn, resulting in less confident predictions^17^. Li et al., also reported higher performance of DeepRisk model using preoperative whole brain MRI without tumor segmentation than that of a ResNet model using accurately segmented tumor images in predicting overall survival of glioma^18^. Therefore, it seems that determination of AI would not be affected by the image crop and even would be biased by crop.

On the other hand, specialists showed significantly better accuracy when tested with cropped images. AI in dental/medical images tends to observe outlines, while specialists are likely to observe specific findings. Moreover, AI uses deep learning algorithms to analyze images and identify patterns, which can lead to the detection of outlines or borders in the images. By contrast, specialists have a deeper understanding of anatomy, pathology, and the human body, so they tend to focus on specific findings or features that are relevant to the diagnosis or treatment of a patient's condition. It is important to note that AI and specialists complement each other in medical imaging: AI can provide a quick and objective analysis of images, while specialists can provide a more in-depth interpretation and understanding of the findings. By combining the strengths of both AI and specialists, medical imaging can be improved, thus leading to better patient outcomes.

The present study has some limitations. First, the data is small and unbalanced due to the low prevalence of DE. To compensate for this, we used a pre-trained model and performed data augmentation. We also selected the best model based on AUC rather than accuracy. When dealing with imbalanced datasets, it is often not sufficient to evaluate a machine learning model based solely on accuracy. This is because accuracy can be misleading in the case of imbalanced datasets, where the number of samples in each class is significantly different. In such cases, a model that always predicts the majority class can have high accuracy, even though it is not useful for classifying the minority class. To address this issue, alternative evaluation metrics such as AUC (Area Under the Receiver Operating Characteristic Curve) can be used. AUC is a metric that measures the performance of a binary classification model across different probability thresholds. It provides an aggregated measure of the model's ability to discriminate between positive and negative samples, regardless of the specific threshold used. In cases of PA where the dataset is imbalanced (normal:abnormal = 119:264), AUC can be a more appropriate evaluation metric than accuracy. A model that only predicts the majority class (i.e., normal) would be expected to have a high accuracy but a low AUC, thus indicating poor performance in classifying the minority class.

Second, we only considered images that were collected from a single institution, rather than a multicenter study. In further research, collecting periapical radiographs from multiple institutions with several different X-ray machines would improve the performance of the model, which would increase the potential utility for clinical applications in the dental field.

Third, DE images included the periapical lesions. Our inclusion criteria for DE involved the presence of periapical lesion or symptoms of pulpitis. This criterion was chosen to specifically target moderate to severe cases of DE that have a significant impact on the prognosis of the affected teeth. There was a possibility that AI’s determination was influenced by the presence or absence of periapical lesion. To investigate this further, activation mapping was conducted, revealing that AI was primarity focused on the coronal part of the dental pulp rather than the periapical area.

Fourth, our inclusion criteria of DE are abnormal cusps observed by the clinician, which does not target worn-down DE. Also, PA is typically taken for teeth that require further investigation, and screening examination are usually performed using panoramic radiography. Therefore, further study to develop a model that can be applied to panoramic radiography or that has a high diagnostic performance for worn-down DE would be necessary.

While AI has shown promise in medical imaging, it is still in its early stages, and it is not yet able to replace human specialists. It is important for AI to be considered as a tool to support and enhance the decision-making process of specialists, rather than as a replacement for their expertise.

## Conclusions

The developed deep learning model showed promising diagnostic capabilities in identifying moderate to severe dens evaginatus using periapical radiography, even with a limited dataset. This algorithm’s effectiveness remained consistent regardless of image segmentation, contrasting with the specialists’ reliance on image cropping to enhance their performance. Consequently, this AI model holds the potential to serve as a reliable and efficient tool for diagnosing dens evaginatus. Its implementation would facilitate early detection, broaden treatment options, and ultimately contribute to improving the long-term prognosis of affected teeth. However, it is important to note that the AI model should complement clinical expertise rather than replace it. Further research is needed to validate and refine the model using larger datasets and explore additional imaging modalities or clinical parameters to enhance diagnostic accuracy.

## Data Availability

The data that support the findings of this study are available from the corresponding authors (Youngdoo Son, youngdoo@dongguk.edu; Min-Seock Seo, profee@naver.com) upon reasonable request.
